# Evaluation of New Calibrated Pulse-Wave Analysis (VolumeView^TM^/EV1000^TM^) for Cardiac Output Monitoring Undergoing Living Donor Liver Transplantation

**DOI:** 10.1371/journal.pone.0164521

**Published:** 2016-10-13

**Authors:** MiHye Park, Sangbin Han, Gaab Soo Kim, Mi Sook Gwak

**Affiliations:** 1 Department of Anesthesiology and Pain Medicine, Kyungpook National University school of Medicine, Daegu, Republic of Korea; 2 Department of Anesthesiology and Pain Medicine, Samsung Medical Center, Sungkyunkwan University School of Medicine, Seoul, Republic of Korea; Universidad de Navarra, SPAIN

## Abstract

**Background:**

Intrapulmonary thermodilution technique using a pulmonary artery catheter is widely used for measuring cardiac output (CO) in patients undergoing liver transplantation. However, its invasiveness and associated complications have led to an interest in less invasive modalities. Thus, we aimed to evaluate whether the new calibrated pulse-wave analysis method monitoring (VolumeView^TM^/EV1000^TM^) is interchangeable with intrapulmonary thermodilution technique.

**Methods:**

Twenty-eight patients undergoing living donor liver transplantation were enrolled in this prospective observational study. COs were recorded automatically by the two devices and compared simultaneously at 10-minute intervals. The agreement of absolute CO values and the tracking ability of CO changes trends were compared. A Bland-Altman analysis with percentage errors and concordance rate for trend analysis using both a 4-quadrant plot and a polar plot were performed on the data.

**Results:**

A total of 375 paired datasets from 25 patients were included in analysis. COs measured by intrapulmonary thermodilution ranged from 3.8–13.7 L/min. The mean CO difference between the two techniques was 0.57 L/min, and the 95% limits of agreement were -0.98 L/min to 2.12 L/min with a percentage error of 42.3%. The percentage errors in the dissection, anhepatic, and reperfusion phase were 30.5%, 31.7%, and 27.4%, respectively. The concordance rate between the two techniques was 78.4%.

**Conclusion:**

The calibrated pulse-wave analysis and intrapulmonary thermodilution failed to show acceptable interchangeability in terms of both estimating CO and tracking CO changes during living donor liver transplantation.

## Introduction

Liver transplantation is associated with frequent changes in the preload and afterload due to surgical clamping and unclamping of major abdominal vessels, sudden blood loss, large fluid administrations, and post-reperfusion syndrome. These surgical events are severely complicated by the underlying circulatory alteration of cirrhotic patients, featuring high cardiac out (CO), low systolic vascular resistance (SVR), tachycardia, and insufficient hemostatic function [1]. Thus, an accurate, continuous, and rapid responsive hemodynamic monitoring method is essential to assess preload, afterload, and cardiac function during liver transplantation. Especially, CO assessment is a key variable in advanced hemodynamic management.

A pulmonary artery catheter (PAC) has been used as the clinical gold standard for assessment of CO during liver transplantation procedures. It is still used to manage patients with significant pulmonary hypertension [2], but we have questions about the risk-benefit ratio of PAC in recipients without pulmonary hypertension who are undergoing living donor liver transplantation (LDLT) [3–6]. LDLT has been developed as an alternative to deceased donor liver transplantation to compensate for a critical shortage of cadaveric organ donations. Thus, recipients are not usually in advanced cirrhotic conditions and the monitoring of pulmonary artery pressure is not essential anymore. Thus, many institutions have tried to decide upon the less invasive method for CO assessment for patients undergoing LDLT.

Recently, a new pulse-wave analysis system (VolumeView^TM^/EV1000^TM^; Edwards Lifesciences, Irvine, CA, USA) has been introduced into clinical practice. It is calibrated through transpulmonary thermodilution (TPTD) and provides a beat to beat real time analysis of the femoral arterial pressure curve. The TPTD performed with this system requires central venous cannula to inject cold saline and a specific thermistor-tipped femoral arterial cannula.

This study evaluated whether the VolumeView/EV1000 system can be used as an alternative to automatic PAC thermodilution for hemodynamic monitoring of patients undergoing LDLT.

## Methods

### Patients

This prospective observational study was approved by the Institutional Review Board of Samsung Medical Center and registered at a public registry (clinicaltrials.gov identifier: NCT02306018). Written informed consent was obtained from 28 consecutive adult patients scheduled for elective adult-to-adult LDLT. Patients with age less than 18 years, contraindication for a femoral artery cannulation, severe valvular heart disease, arrhythmias, intracardiac shunt, or pulmonary hypertension were excluded.

### Study protocols

According to our institutional liver transplantation protocol, invasive hemodynamic monitoring techniques were performed via the radial artery, femoral artery, femoral vein, and internal jugular vein after induction. We used a VolumeView catheter (diameter: 4 Fr, length 16 cm; Edwards Lifesciences, LLC, Irvine, CA) that was introduced into the right femoral artery and connected to the EV1000 system, which includes a panel interface and data box (Edwards Lifesciences, software version 1.0).

A PAC (Swan-Ganz CCOmbo V, Edwards Lifesciences, LLC, Irvine, CA) was introduced through the right internal jugular vein in combination with a 9-Fr large-bore central venous catheter and threaded into the pulmonary artery. The position of the PAC tip was adjusted to detect waveforms of pulmonary artery pressure and provide a signal quality indicator throughout the surgery. The PAC was connected to a Vigilance^TM^ system monitor (Edwards Lifesciences, LLC, Irvine, CA) to obtain CO measured by STAT-mode. The STAT-mode provides CO measurements that are time-averaged over the preceding 1 minute.

After placement of the catheters in the femoral and pulmonary artery, all time clocks on the EV1000 and Vigilance monitors were synchronized. The TPTD measurement using the VolumeView/EV1000 system use a bolus injection through a central venous catheter situated above the diaphragm and a femoral arterial catheter with a specific thermistor tip subsequently measures the thermodilution curve. The TPTD measurement for VolumeView/EV1000 was performed in sets of two or three bolus injections of 20 ml cold isotonic saline through the central venous catheter irrespective of the ventilator cycle. The results were accepted to show a consistent shape of the thermodilution curve and had less than 15% variation from previous measurements. The intermittent bolus TPTD measurements for calibration were performed at specific four times: after induction, after retractor placement in the abdominal wall, after portal vein clamping, and 10 min after reperfusion. To avoid variation, the injection was always performed by the same person when the patient was in a stable hemodynamic condition (mean arterial pressure ≥ 70 mm Hg and sinus rhythm) with a constant rate of intravenous fluid infusion and drugs. And surgical procedures were paused. Between TPTD calibrations, the VolumeView/EV1000 system uses a hybrid CO algorithm for continuous CO (CO_FA_) measurements:

$${CO}_{FA}=CO\;(TPTD)\;*\;f\;(\Delta \;conventional\;femoral\;artery\;pulse\;contour\;parameters,\;\Delta \;advanced\;femoral\;artery\;waveshape\;parameters)$$

Tone and flow changes are determined by assessing the relationship between conventional pulse contour and advanced waveshape parameters. Conventional pulse-wave parameters are determined considering the fundamental work of Wesseling assuming that only the area under the systolic part of the pressure waveform is related to stroke volume by aortic impedance. And advanced arterial pressure waveshape parameters are derived from analysis of the pressure waveform of the entire heart cycle [7,8].

After induction (T0), we compared TPTD CO using VolumeView/EV1000 to PAC CO using STAT-mode automatic PAC thermodilution to evaluate the agreement of thermodilution between the two techniques. During each surgical procedure, 15 sets of measurements per individual were obtained at 5, 15, 25, 35, and 45 min after re-calibration by TPTD; after retractor placement in the abdominal wall (T1-T5), after portal vein clamping (T6-T10), and after reperfusion (T11-T15). Hemodynamic data were grouped into three phases: dissection phase (T1-T5), anhepatic phase (T6-T10), and reperfusion phase (T11-T15).

### Anesthetic management and surgical procedures

Non-invasive patient monitoring included 5-lead electrocardiography, pulse oximetry, noninvasive arterial blood pressure, and end-tidal CO_2_. Anesthesia was induced with thiopental sodium at 5 mg/kg, vecuronium at 1.5 mg/kg, and sevoflurane. All of the patients were intubated with endotracheal tubes. Patients were mechanically ventilated with a tidal volume of 8 ml/kg in 50% oxygen using medical air at a fresh gas flow rate of 2 L/min. The respiratory rate was adjusted as needed to maintain normocapnia. Anesthesia was maintained by isoflurane titrated to a bispectral index below 60 and vecuronium at 0.8–1.0 mcg/kg/min. Remifentanil was infused intravenously at the rate of 0.02–0.20 mcg/kg/min according to hemodynamic responses. Following our liver transplantation anesthetic management protocol, we aimed to maintain a mean arterial pressure ≥ 70 mm Hg by infusions of fluids and rescue drugs, such as dopamine and norepinephrine. Body temperature was kept above 35°C by using a warming blanket, heat and moisture exchanger, room temperature thermostatically set at 24°C, vinyl arm wraps, and a rapid fluid warmer.

Graft implantation was performed using a piggyback technique to preserve the caval flow by partial inferior vena cava (IVC) clamping during the anastomosis of the hepatic vein with the IVC. After portal vein anastomosis, the graft was reperfused by consecutively unclamping the hepatic vein and portal vein. Subsequently, hepatic artery anastomosis was performed, followed by biliary anastomosis. The PAC was removed from the patients at the end of surgery in the operating room, if there had been no specific hemodynamic events.

### Statistical analysis

Statistical calculations were performed using SPSS 20.0 (SPSS Inc., Chicago, IL, US) and R 3.0.3 (Vienna, Austria). We compared the CO of the two methods using correlation analysis and Bland-Altman analysis. We compare Baseline TPTD CO measurements using Volumeview/EV1000 versus PAC thermodilution, and the continuous CO of Volumeview/EV1000 (CO_FA_) versus continuous CO of PAC (CO_PAC_) at all time points were also compared. Bland-Altman analysis was used to assess the agreement between two techniques. Bias, limits of agreements, and percentage error between the two techniques were calculated. We adjusted for the effects of repeated measurements within each subject in the Bland-Altman analysis using the method suggested by Myles and Cu [9,10]. The percentage error (1.96 * standard deviation (SD) / mean CO) was calculated with 30% taken as clinically acceptable. For calculation of SD, the data were analyzed with a linear mixed effects model. To assess the trending ability, we used two different methods, the four-quadrant plot and the polar plot. Changes in serial CO readings were calculated by subtracting consecutive CO readings. Data at the center of the plot tend to be statistical noise, a central exclusion zone of 10% of mean CO or ±1.0 L/min for small changes in CO was applied [6,11]. The concordance rate was defined as the percentage of the total number of points in the lower left or upper right quadrant of the four quadrant plot, which is considered to be good when it exceeds 92%. The polar-plot analysis was used to assess angular concordance rate (the percentage of points within a ±30° radial zone), angular bias (the average angle from the axis), and radial limits of agreement (the radial zone containing 95% of the total number of data points). The agreement between the reference and tested methods is regarded as excellent when the following limits are met: (1) angular concordance rate more than 95%, (2) mean angular bias within ±5°, and (3) radial limits of agreement within ±30°, as indicated in a previous study [12]. We also separately analyzed the three phases (dissection, anhepatic, and reperfusion) with a Bland-Altman analysis with percentage error and concordance rate.

The associations between the distance in CO between the two techniques and the indices that were measured from PAC were analyzed using Pearson’s correlation. Statistical significance was set at a *P*-value < 0.05.

## Results

This study was performed in LDLT patients undergoing planned surgeries between December 2014 and September 2015. We enrolled 28 patients, and data from 25 patients were analyzed. One patient was not studied because metastatic peritoneal seeding was found during surgery. A PAC could not be placed in one patient, we tried to insert catheter only on the right side. And the VolumeView catheter could not be placed in one patient. The femoral artery was easily punctured with 20-gauge needle, but the VolumeView catheter could not be advanced through the artery. Instead smaller 20-gauge leader catheter (diameter: 3 Fr, length 8 cm; Vygon) was inserted. No complications related to the VolumeView catheter were observed during this study. The demographic data and principal diagnoses are listed in Table 1. All recipients showed sinus cardiac rhythm and a preoperative peak in right ventricular pressure of ≤ 40 mm Hg.

**Table 1:** Preoperative and intraoperative clinical data of liver transplantation recipients.

Characteristic	Descriptive statistics
Age (years)	54 ± 9 (35–68)
Female/male	7/18
Body mass index (kg/m^2^)	20.7 ± 2.4 (16.3–24.5)
Underlying disease	
HBV related hepatocellular carcinoma with cirrhosis	11
HBV related hepatocellular carcinoma without cirrhosis	3
HBV cirrhosis	5
Alcoholic cirrhosis	1
Cryptogenetic cirrhosis	2
Autoimmune cirrhosis	2
Wilson disease	1
Child classification (A/B/C)	11 /8 /6
MELD score	15 ± 8 (6–35)
<10	7
10–19	12
20–29	4
≥30	2

Data are described as mean ± standard deviation (range) or number.

HBV = Hepatitis B virus, MELD = Model for End-Stage liver disease

CO measurements had a broad range of 3.8–13.7 L/min derived from PAC and 3.6–13.5 L/min derived from VolumeView/EV1000. CO, SVR, and temperature measurements derived from the two methods at all time points and phases are reported in Tables 2 and 3.

**Table 2:** Correlation coefficient, bias, and limits of agreement of all measurements and time points.

Time	Intra Correlation coefficient	Bias (L/min)	Limits of agreement (L/min)	CO_PAC_ (L/min)	CO_FA_ (L/min)	SVR_PAC_ (dynes. s. cm^5^)	SVR_FA_ (dyne. s. s. cm^5^)	BT (°C)
T0	0.940	0.33	-0.93 to 1.59	5.8±1.2	5.5±1.2	1055±286	1089±298	35.8±0.4
T1	0.939	0.44	-1.28 to 2.16	7.1±2.0	6.6±1.6	910±244	934±260	35.8±0.4
T2	0.920	0.28	-1.66 to 2.21	7.1±2.0	6.8±1.6	936±284	913±245	35.9±0.4
T3	0.926	0.50	-1.34 to 2.33	7.3±1.9	6.8±1.7	1012±319	931±239	35.9±0.4
T4	0.914	0.51	-1.43 to 2.45	7.4±1.8	6.9±1.7	977±289	964±273	36.0±0.4
T5	0.945	0.64	-1.12 to 2.40	7.6±2.0	7.0±1.9	937±269	946±270	36.0±0.3
T6	0.928	0.15	-1.65 to 1.96	7.0±1.7	6.8±1.9	953±252	960±235	36.1±0.4
T7	0.927	0.54	-1.26 to 2.33	7.2±1.9	6.5±1.7	931±253	941±241	36.1±0.4
T8	0.945	0.64	-0.99 to 2.28	7.1±2.0	6.4±1.8	867±253	971±238	36.0±0.5
T9	0.944	0.40	-1.33 to 2.12	6.8±2.1	6.4±1.8	942±282	952±228	36.0±0.5
T10	0.970	0.46	-0.75 to 1.66	6.8±1.9	6.4±1.7	931±270	952±244	36.1±0.5
T11	0.914	0.86	-0.89 to 2.60	8.0±1.6	7.2±1.6	720±176	717±218	36.0±0.5
T12	0.921	0.62	-1.27 to 2.51	8.1±1.7	7.5±1.5	702±193	736±232	36.0±0.5
T13	0.929	0.83	-0.73 to 2.40	8.2±1.6	7.4±1.5	670±154	724±190	36.0±0.5
T14	0.929	0.96	-0.78 to 2.71	8.1±1.7	7.1±1.8	647±147	718±185	36.1±0.5
T15	0.868	0.70	-1.45 to 2.85	8.0±1.6	7.3±1.6	661±172	706±201	36.1±0.5

Data are shown as mean ± standard deviation

COPAC = Pulmonary artery catheter cardiac output, COFA = VolumeView/EV1000 cardiac output

SVRPAC = Pulmonary artery catheter systemic vascular resistance

SVRFA = VolumeView/EV1000 systemic vascular resistance, BT = Body core temperature

**Table 3:** Intraoperative measurements are presented separately for each surgical phase.

	Total		Dissection		Anhepatic		Reperfusion	
	PAC	VolumeView	PAC	VolumeView	PAC	VolumeView	PAC	VolumeView
CO (L/min)	7.5 (1.9)	6.9 (1.7)	7.3 (1.9)	6.8 (1.7)	7.0 (1.9)	6.6 (1.7)	8.1 (1.6)	7.3 (1.6)
Range	3.8–13.7	3.9–13.5	4.4–13.7	3.9–11.8	3.8–12.3	4.0–13.5	5.0–12.9	4.1–12.6
SVR (dyne.s.cm^5^)	853 (275)	871 (254)	955 (280)	937 (254)	925 (273)	955 (234)	680 (169)	720 (203)
Range	349–1923	413–1582	349–1693	429–1582	424–1923	433–1547	351–1174	413–1381

Data are mean (standard deviation)

PAC = Pulmonary artery catheter, VolumeView = VolumeViewTM/EV1000TM

CO = Cardiac output, SVR = Systemic vascular resistance, SV = Stroke volume

After induction (T0), the baseline TPTD CO using Volumeview/EV1000 and PAC CO showed close agreement between the two methods. Bias between TPTD CO and PAC CO was -0.3 L/min, and 95% limits of agreement were -0.9 to 1.5 L/min with a percentage error 22.8%. Baseline mean CO and SVR derived from PAC was 5.8 L/min (range, 4.0 to 8.6) and 1055 dynes.s.cm^5^ (range, 568 to 1759). Most recipients showed normal range CO and SVR before starting the surgical procedure.

**Fig 1:**
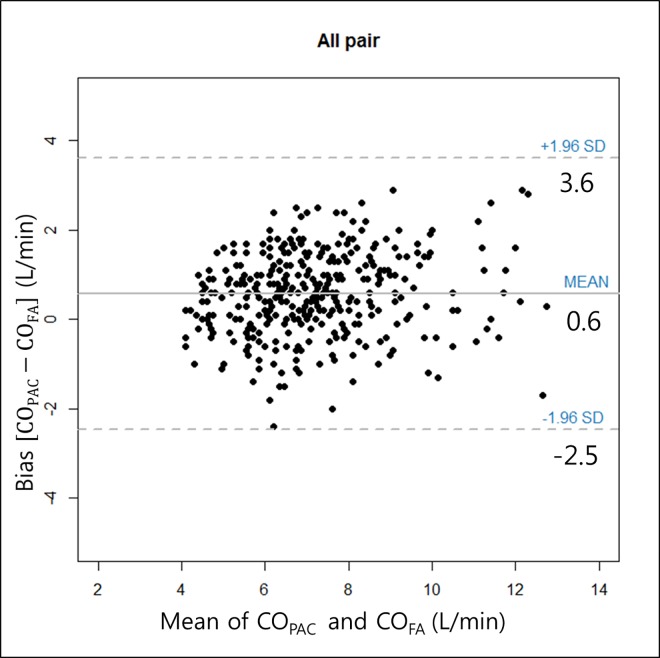
Bland-Altman plots for all 375 data comparisons in 25 patients. Bias and limits of agreement (±1.96 SD) are shown in the plot.

**Fig 2:**
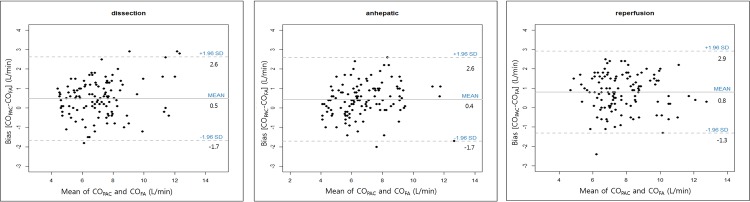
Bland-Altman analysis with repeated measurements using random effects modeling percentage errors (dissection /anhepatic /reperfusion phase). Bias and limits of agreement (±1.96 SD) are shown in the plots.

During the surgical procedures, a total of 375 paired data sets were ultimately included. The overall mean CO_PAC_ was 7.5 ± 1.9 L/min (range, 3.8 to 13.7), which was slightly higher than the overall mean CO_FA,_ 6.9 ± 1.7 L/min (range, 3.9 to 13.5). A good correlation coefficient (r^2^ = 0.76, *P* = 0.00) was found for the CO_PAC_ and CO_FA_ comparison. The Bland-Altman analysis data were corrected for repeated measures using random effects modeling, after which the mean bias (CO_PAC_-CO_FA_) became 0.57 L/min and the adjusted limits of agreement became -2.47 to 3.60 L/min (Fig 1). The adjusted percentage error was 42.3%. The proportion of paired datasets with < 1.0 L/min difference was 77.0%. A total of 375 data sets were divided into three subgroups according to the operative phase (125 data sets for each phase). CO_PAC_ and CO_FA_ showed agreement slightly above the limits of clinical acceptance, a percentage error of 30.5% during the dissection phase, and 31.7% during the anhepatic phase. However, an acceptable agreement with a percentage error of 27.4% was observed after graft implantation (Fig 2).

**Fig 3:**
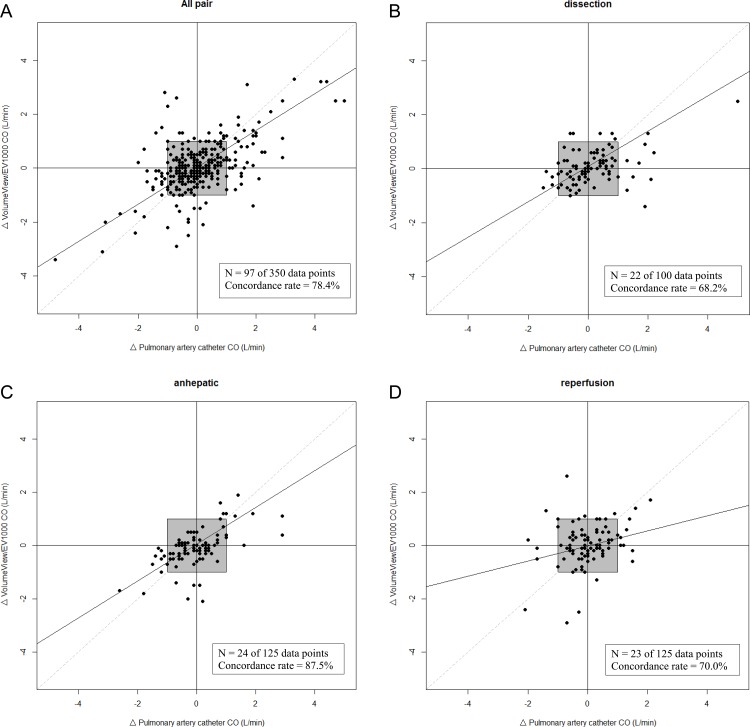
Four quadrant scatter plot comparing changes in the VolumeView/EV1000 and pulmonary artery catheter cardiac output readings. Data points within the ± 1.0 L/min exclusion zone (gray box) are excluded from analysis.

Four quadrant plots of the CO changes were drawn (Fig 3). A central exclusion zone for small CO changes was set at ± 1.0 L/min. There were 350 data pairs that compared CO_PAC_ changes with CO_FA_ changes throughout surgery, and these were reduced to 97 data pairs after exclusion of central zone data. The concordance rate between the two techniques was 78.4%. The concordance rate in the dissection, anhepatic, and reperfusion phase were 68.2%, 87.6%, and 70.0%, respectively.

**Fig 4:**
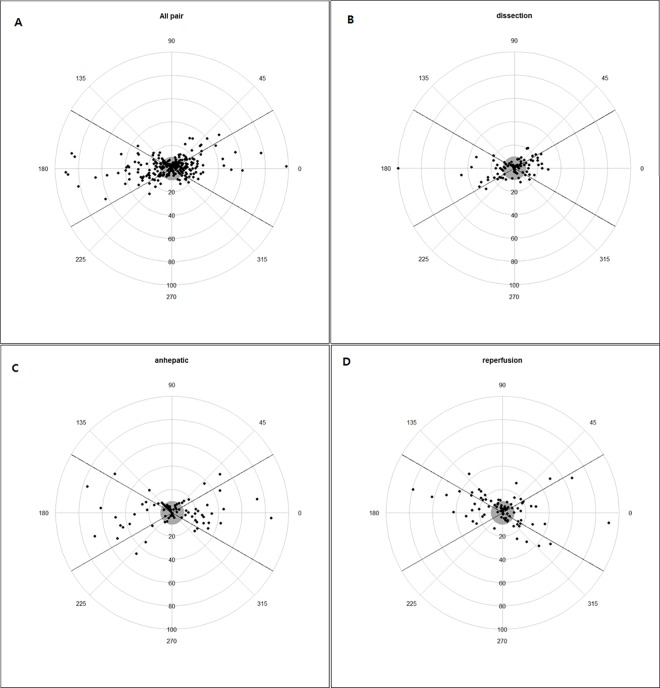
Polar plot comparing changes in the VolumeView/EV1000 and pulmonary artery catheter cardiac output readings. Data points within mean changes ≤10% exclusion zone (gray circle) were excluded from the analysis.

The polar-plot analyses are shown in Fig 4. The angular concordance rates were 69.8%, 62.2%, 62.5%, and 53.3%; the angular biases were 5.6°, 6.8°, 0.6° and 9.4°; and the radial limits of agreement were -55.3° to 55.7°, -40.8° to 60.2°, -38.8° to 47.5° and -44.3° to 54.0° in all pairs, dissection, anhepatic, and reperfusion, respectively.

The distance in CO between the two techniques was positively correlated with CO (Pearson coefficient *r* = 0.399, *P* < 0.001), and Model for End-Stage liver disease score (*r* = 0.129, *P* = 0.013). That was negatively correlated with SVR (*r* = -0.342, *P* < 0.001) and body core temperature (*r* = -0.116, *P* = 0.025).

## Discussion

This study demonstrates that the VolumeView/EV1000 system using a new calibrated pulse-wave analysis algorithm is not interchangeable with established automatic thermodilution via PAC in patients undergoing LDLT. The percentage error from our Bland-Altman analysis was 42.3% when adjusted for repeated measures. The concordance rate was 78.4% when ± 1.0 L/min central zone data were excluded. In the present study, better agreement and comparable trending capability than other pulse-wave analysis methods were observed [13–16], but these values exceed those recommended as being clinically acceptable in order for two clinical techniques to be considered as interchangeable.

Although CO measurements of intermittent PAC thermodilution have been considered the clinical gold standard, continuous CO monitoring is preferable to intermittent CO monitoring for informing management strategies [17,18]. But an insertion of PAC has potential risk for severe complications, such as arrhythmias, pulmonary artery injury, right ventricular perforation [19], thrombosis[20], and infection. Especially, we have experienced liver allograft recipients have a relatively high incidence of severe ventricular arrhythmias are more often associated with mechanical irritation or trauma of the endocardium than with other patient factors [21]. Thus, less invasive continuous CO monitoring techniques are desperately required during LDLT.

Pulse wave analysis systems have offered less invasive, continuous, operative-independent, and quick-use systems for monitoring of hemodynamic parameters [22]. And they have provided useful indices of volume status based on the shape of the measured waveform to predict fluid responsiveness during liver transplantation [23]. However, their CO measurements showed insufficient precision and limited reliability [14–16,24].

The FloTrac/Vigileo (Edwards Lifesciences, LLC, Irvine, CA) system is based on pulse pressure analysis, but does not require an external reference method for calibration. It was widely evaluated in cirrhotic patients undergoing liver transplantation. However, inaccurate measurements were observed because of variations in SVR, it was still not in acceptable agreement and trending with PAC-derived measurements [14,15]. Unlike the VolumeView/EV1000 system, the Flotrac/Vigileo system was an adjusted uncalibrated artery waveform and usually obtained the signal from a radial artery site. In cirrhotic patients, low vascular tone or high dose vasoconstrictor drugs affect the precision of arterial wave-form analysis. Thus, external calibration for adjusting vascular tone change and central arterial pressure wave forms might be needed to assess CO during liver transplantation [24–26]. While several consider femoral artery catheterization invasive, the risks associated with the arterial catheter are low [27] and no adverse effects of the VolumeView catheter were observed in this study. Among calibrated central arterial pressure based monitor, the PiCCO system (Pulsion Medical System, Munich, Germany) was studied in cirrhotic patients. It has been tested in operative and intensive care unit settings, but conflicting results have been reported [28,29].

Like PiCCO system, VolumeView/EV1000 system uses two mechanisms. The TPTD measurement for the calibration of the system is required to adjust for aortic impedance, which differs from patient to patient. The TPTD technique used has already been confirmed to have good agreement with thermodilution via PAC in many animals and clinical studies [24,28,30]. In the present study, the agreement of baseline measurements was good, but the value of TPTD CO by VolumeView/EV1000 was lower than PAC CO. The value of TPTD is generally thought to be greater than the corresponding intermittent PAC thermodilution, because the value of PAC is a measure of right ventricular output whereas TPTD measures left ventricular output. On the other hand, previous studies also demonstrated continuous PAC thermodilution overestimates compared to intermittent PAC thermodilution [31]. We did not perform intermittent PAC thermodilution, so it is not clear which is the true value of CO. Thus, we could not determine the mechanism of the underestimation of TPTD in this system.

Between TPTD calibrations, changes in tone are assessed by alterations in femoral artery wave shape variables. The femoral pulse-wave is calibrated by TPTD, this hybrid CO algorithm uses for continuous CO monitoring. Bendjelid et al. found that in critically ill patients and across a wide range of clinical situations the VolumeView/EV1000 system performed as accurately as the PiCCO system, and an improved precision was observed for the VolumeView technique [7]. The improvement process of the VolumeView/EV1000 system includes an additional consideration of an advanced pressure waveform analysis. For accurate measurements, a reliable detection of both the systolic and the diastolic waveform portion are assessed. This may better reflect the actual conditions of the cardiovascular system in cirrhotic patients [32]. Thus, we expected CO using the VolumeView/EV1000 system to improve precision relative to other pulse-wave analysis methods during liver transplantation.

However, the results of the present study were poorer than expected from the improvement of algorithm. We assume this is due to several reasons. First, the individual aortic impedance can be affected at several points during the procedure as a result of direct manipulation of the large abdominal vessels. It is difficult to obtain accurate femoral arterial waveforms at these times. Second, prior results show that calibrated pulse-wave analysis can be affected by body core temperatures, SVR, and vasoconstrictor drugs. The accuracy and precision of measuring CO by thermodilution were markedly decreased after portal vein clamping, because of changing body core temperature and vasoconstrictor [31,33]. Third, there were the limitations of statistical analysis. The clinical acceptance of agreement with a percentage error of 30% was not achieved. However, high cardiac output and hemodynamic instability were present throughout the LDLT procedures. According to Peyton et al., the level of precision of agreement remains well outside the 30% limits across a range of patient groups and clinical situations. They suggest, based on their empirical findings, a percentage error in agreement with thermodilution of ±45% presents a more realistic expectation of achievable precision in clinical practice [34]. However, during surgery, reliable real-time tracking of the direction of changes in CO is arguably more important than the ability of the monitor to deliver a highly accurate single measurement under stable conditions. In this study, the tracking change between two techniques showed limited results.

This study has several limitations. First, we compared VolumeView/EV1000 to CO measured by STAT-mode automatic PAC thermodilution. Intermittent thermodilution has pitfalls related to operator variation, patient pathologies, and temperature. Also, acceptable limits of agreement of intermittent thermodilution and continuous automatic thermodilution via PAC have already been shown in previous studies [31,35]. And the aim of our study was to determine whether the new device is interchangeable with continuous PAC thermodilution. Second, we did not test the techniques’ performance after any intervention such as fluid challenge and drugs administration that might influence the hemodynamic status. Thus, the real tracking ability of VolumeView/EV1000 has not been completely evaluated.

In conclusion, the VolumeView/EV1000 system showed better agreement and comparable trending capability than other less invasive method were observed during living donor liver transplantation. However, it is not yet interchangeable with automatic PAC thermodilutionin cirrhotic patients during liver transplantation.

## Supporting Information

S1 File A dataset for the present study. (ZIP)

S1 Text Study protocol. (DOCX)

S2 Text Intraoperative hemodynamic parameters. (DOCX)
