# Supplementary material for: Evaluation of New Calibrated Pulse-Wave Analysis (VolumeViewTM/EV1000TM) for Cardiac Output Monitoring Undergoing Living Donor Liver Transplantation
Source: PLoS One. 2016 Oct 13;11(10):e0164521. doi: 10.1371/journal.pone.0164521 (PMC5063283; doi:10.1371/journal.pone.0164521)
Supplement: S1 Text — (DOCX) [file pone.0164521.s002.docx]

S 1 Text. Study protocol

After general anesthesia induction (pulmonary artery catheter (PAC) insertion)

**↓**

EV1000 calibration through TPTD

Compared PAC CO and EV1000 TPTD (T0) : Bland-Altman analysis

**↓**

after retractor placement in the abdominal wall, performed recalibration by TPTD

5 minutes after TPTD

Dissection phase

PAC CO (CO_PAC_) and EV1000 CO (CO_FA_) were recorded for 5 times with 10 minutes interval (T1-T5)

: Bland-Altman analysis (Values), four quadrant plot (changes)

**↓**

after portal vein clamping (T6-T10), performed recalibration by TPTD

Anhepatic phase

5 minutes after TPTD

PAC CO (CO_PAC_) and EV1000 CO (CO_FA_) were recorded for 5 times with 10 minutes interval (T6-T10)

: Bland-Altman analysis (Values), four quadrant plot (changes)

**↓**

after reperfusion, performed recalibration by TPTD

Reperfusion phase

5 minutes after TPTD

PAC CO(CO_PAC_) and EV1000 CO(CO_FA_) were recorded for 5 times with 10 minutes interval (T11-T15)

: Bland-Altman analysis (Values), four quadrant plot (changes)

T1~T15 dataset compared Bland-Altman analysis (Values) and four quadrant plot (changes).

<Evaluation of new calibrated pulse-wave analysis (VolumeView/EV1000)

for cardiac output monitoring undergoing liver transplantation> investigator confirm :

Case Code : date: gender: height/weight: MELD score:

| TIME |  | Pulmonary artery catheter | | | | |  | VolumeView/EV1000 | | | |
| --- | --- | --- | --- | --- | --- | --- | --- | --- | --- | --- | --- |
| T0 | BP | BT | CO/CI | SVR | SV | EF |  | CO/CI | SVR | SV | SVV |
| T1 |  |  |  |  |  |  |  |  |  |  |  |
| T2 |  |  |  |  |  |  |  |  |  |  |  |
| T3 |  |  |  |  |  |  |  |  |  |  |  |
| T4 |  |  |  |  |  |  |  |  |  |  |  |
| T5 |  |  |  |  |  |  |  |  |  |  |  |
| T6 |  |  |  |  |  |  |  |  |  |  |  |
| T7 |  |  |  |  |  |  |  |  |  |  |  |
| T8 |  |  |  |  |  |  |  |  |  |  |  |
| T9 |  |  |  |  |  |  |  |  |  |  |  |
| T10 |  |  |  |  |  |  |  |  |  |  |  |
| T11 |  |  |  |  |  |  |  |  |  |  |  |
| T12 |  |  |  |  |  |  |  |  |  |  |  |
| T13 |  |  |  |  |  |  |  |  |  |  |  |
| T14 |  |  |  |  |  |  |  |  |  |  |  |
| T15 |  |  |  |  |  |  |  |  |  |  |  |
|  |  |  |  |  |  |  |  |  |  |  |  |

BP:blood pressure(femoral artery),BT:body temperature(pulmonary artery catheter), CO/I:cardiac output/index, SVR:systenuc vascular resistance, SV:stroke volume, EF:ejection fraction, SVV:stroke volume variation
