# Supplementary material for: Evaluation of New Calibrated Pulse-Wave Analysis (VolumeViewTM/EV1000TM) for Cardiac Output Monitoring Undergoing Living Donor Liver Transplantation
Source: PLoS One. 2016 Oct 13;11(10):e0164521. doi: 10.1371/journal.pone.0164521 (PMC5063283; doi:10.1371/journal.pone.0164521)
Supplement: S2 Text — (DOCX) [file pone.0164521.s003.docx]

|  | HR (bpm) | MBP(mmHg) | CVP | SV_PAC_ | SV_volumeView_ | SVV_volumeview_ |
| --- | --- | --- | --- | --- | --- | --- |
| T1 | 89.3 (14.9) | 85.7 (10.3) | 7.6 (2.6) | 77.8 (22.5) | 76.4 (20.5) | 6.8 (3.4) |
| T2 | 86.9 (13.2) | 81.6 (11.8) | 7.2 (2.8) | 80.6 (21.3) | 79.1 (22.4) | 7.0 (3.4) |
| T3 | 87.5 (10.0) | 81.5 (11.5) | 7.6 (2.6) | 81.4 (22.6) | 79.5 (23.6) | 7.0 (3.6) |
| T4 | 86.7 (11.2) | 85.6 (12.3) | 7.3 (2.4) | 82.2 (20.2) | 78.8 (23.0) | 7.6 (4.4) |
| T5 | 88.3 (11.2) | 83.4 (8.6) | 7.2 (2.2) | 82.3 (21.7) | 79.5 (24.8) | 7.8 (4.1) |
| T6 | 93.0 (14.4) | 84.5 (8.9) | 7.1 (2.6) | 75.8 (17.7) | 73.8 (19.5) | 10.3 (7.2) |
| T7 | 91.0 (13.2) | 81.4 (10.4) | 6.9 (2.6) | 78.6 (20.3) | 74.6 (18.7) | 8.6 (4.9) |
| T8 | 91.5 (14.3) | 79.2 (8.7) | 7.0 (2.3) | 79.2 (22.3) | 70.4 (19.2) | 9.7 (5.3) |
| T9 | 91.7 (15.6) | 79.7 (10.5) | 6.8 (2.4) | 75.2 (22.5) | 70.4 (20.0) | 8.9 (4.2) |
| T10 | 92.2 (15.2) | 79.0 (7.9) | 6.6 (2.5) | 74.4 (22.9) | 70.6 (20.9) | 9.5 (4.1) |
| T11 | 93.3 (13.0) | 78.2 (9.6) | 8.6 (2.8) | 84.0 (15.5) | 77.6 (16.7) | 7.2 (3.9) |
| T12 | 93.0 (12.8) | 75.8 (10.1) | 8.0 (2.7) | 87.5 (17.6) | 81.2 ( 17.9) | 6.4 (3.8) |
| T13 | 92.2 (12.8) | 75.1 (10.4) | 8.0 (2.6) | 88.4 (16.9) | 80.2 (14.0) | 7.0 (3.6) |
| T14 | 93.4 (13.5) | 73.4 (8.0) | 8.1 (2.6) | 87.7 (16.0) | 77.5 (16.1) | 7.5 (2.8) |
| T15 | 95.1 (13.1) | 73.9 (7.1) | 8.0 (2.7) | 87.0 (17.1) | 77.5 (16.6) | 7.2 (3.0) |

S2 Text. Intraoperative hemodynamic parameters

Data are mean (SD)

PAC= Pulmonary artery catheter, VolumeView = VolumeView/EV1000

HR = Heart rate, MBP = Mean blood pressure, CVP = Central venous pressure,

SV = Stroke volume, SVV = Stroke volume variation
